# An empirical Bayesian approach to incorporate directional movement information from a forage fish into the Arnason-Schwarz mark-recapture model

**DOI:** 10.1186/s40462-021-00241-1

**Published:** 2021-02-24

**Authors:** Mary A. Bishop, Jordan W. Bernard

**Affiliations:** 1 Prince William Sound Science Center, 300 Breakwater Ave, Cordova, 99574 AK USA; 2 current address: Alaska Department of Fish and Game, 1300 College Rd, Fairbanks, 99701 AK USA

**Keywords:** Arnason-Schwarz, Mark-recapture, Cormack-Jolly-Seber, Bayesian, Acoustic telemetry, Partial migration, Herring, Prince William Sound

## Abstract

**Background:**

Over the past two decades, various species of forage fish have been successfully implanted with miniaturized acoustic transmitters and subsequently monitored using stationary acoustic receivers. When acoustic receivers are configured in an array, information related to fish direction can potentially be determined, depending upon the number and relative orientation of the acoustic receivers. However, it can be difficult to incorporate directional information into frequentist mark-recapture methods. Here we show how an empirical Bayesian approach can be used to develop a model that incorporates directional movement information into the Arnason-Schwarz modeling framework to describe survival and migration patterns of a Pacific herring (*Clupea pallasii*) population in coastal Alaska, USA.

**Methods:**

We acoustic-tagged 326 adult Pacific herring during April 2017 and 2018 while on their spawning grounds in Prince William Sound Alaska, USA. To monitor their movements, stationary acoustic receivers were deployed at strategic locations throughout the Sound. Receivers located at the major entrances to the Gulf of Alaska were arranged in parallel arrays to determine the directional movements of the fish. Informative priors were used to incorporate the directional information recorded at the entrance arrays into the model.

**Results:**

A seasonal migratory pattern was found at one of Prince William Sound’s major entrances to the Gulf of Alaska. At this entrance, fish tended to enter the Gulf of Alaska during spring and summer after spawning and return to Prince William Sound during the fall and winter. Fish mortality was higher during spring and summer than fall and winter in both Prince William Sound and the Gulf of Alaska.

**Conclusions:**

An empirical Bayesian modeling approach can be used to extend the Arnason-Schwarz modeling framework to incorporate directional information from acoustic arrays to estimate survival and characterize the timing and direction of migratory movements of forage fish.

## Background

Abundant, and occurring in large schools, forage fish comprise 20–30% of the global fish catch [1]. These small fish also play a key role in the pelagic food web by transferring energy from primary or secondary producers to a wide variety of higher trophic level predators including seabirds, marine mammals, and other fish species [2]. Despite their importance in the marine ecosystem, little is known about the movement of forage fish. Because of their small size, and in some species the sensitivity to handling [3], most movement studies of forage fish have relied on fishery-dependent methods including traditional mark-recapture [4, 5] or catch-per-unit effort (cpue) [6] analyses. However, with characteristically low recapture rates (e.g. <1% [5]) data from traditional mark-recapture and cpue methods usually lack the temporal and spatial resolution to understand the timing and amount these fish move.

Knowing more about the movements of forage fish would help us better understand their population dynamics, food webs, and provide for better fisheries management. Forage fish often make long migrations between their spawning, foraging and wintering grounds. These migration patterns can shift due to multiple external factors including population collapses and recoveries, prey distributions, abiotic conditions, and climate [7–11], impacting predator populations as well as commercial fisheries management [10–12]. For example, commercially important capelin (*Mallotus villosus*) stocks in the North Atlantic have regularly experienced population collapses. Following the 1991–92 collapse of the Newfoundland stock, capelin became less migratory and remained inshore year-round. With the shift to inshore waters, capelin stocks were closer to seabird colonies and became a significant proportion of seabird diets [11]. In the case of Norwegian spring-spawning herring (*Clupea harengus*), the world’s largest commercially fished herring stock, over a 60-year period, spawning, feeding, and wintering migration routes and locations have changed [12, 13]. The establishment of new and discrete wintering ground locations in this herring population have been attributed to numerically dominant first-time spawners when the social learning process was disrupted due to high fishing pressure on and a scarcity of older herring [10, 12, 14].

Pacific herring (*C. pallasii*) in the North Pacific Ocean is a widely distributed pelagic forage fish that supports important commercial fisheries. In Alaska’s Prince William Sound (Sound), the herring population historically supported five fisheries, representing an annual commercial harvest of up to 20,000 metric tonnes [15]. In 1989 the *Exxon Valdez*, an oil tanker, ran aground in the Sound and spilled approximately 35,000 metric tonnes of crude oil. Four years after the spill, the Sound’s adult herring population collapsed and has yet to recover [16, 17]. Conservation concerns about the lack of recovery of the Sound herring population make it increasingly important to document migration patterns to improve our understanding of adult herring survival [3]. Two important knowledge gaps for the Sound herring population are where post-spawning adults go to feed during the summer and fall and where they overwinter. Elsewhere after spawning, Pacific herring may feed and overwinter in different areas separated by as much as 1000s of kilometers. And within a spawning aggregation, herring migration patterns can vary by local populations with both resident and migratory adult Pacific herring often occurring within the same stock [18], a phenomenon known as partial migration [19].

Over the past two decades miniaturized acoustic transmitters that emit ultrasonic signals have been used successfully to study movements and survival in forage fish species including juvenile salmon (*Oncorhynchus spp.*) [20, 21], adult capelin [22], and adult herring (*Clupea spp.*) [3, 23]. Passive acoustic telemetry has numerous advantages over traditional mark-recapture methods in that fish can be repeatedly located without being physically recaptured. With this tracking method, when a tagged fish swims within the detection range of a submerged hydrophone receiver, an encoded identification and date/time stamp are recorded by the receiver. While mobile acoustic receivers can be used for active tracking, more often acoustic receivers are positioned in stationary grid formations or in a line of receivers deployed as a curtain for continuous, passive monitoring [24].

Methods to analyze individual fish movements based on detections from spatial arrays have ranged from interpolation among locations of receivers [25–27], to state-space models [28, 29]. Another approach is the Arnason-Schwarz (AS) [30] model which generalizes the Cormack-Jolly-Seber mark-recapture model [31–33] so that multiple geographic locations can be accounted for. AS models, including Bayesian versions of the model, have been used to examine animal migration by describing the rates at which animals transition between geographic regions while accounting for mortality and state uncertainty [34–37]. However, a major shortcoming of these techniques for movement analyses when applied to spatial arrays is that information related to the direction of animal movement is not incorporated, which increases state uncertainty.

Incorporating directional movement reduces state uncertainty and therefore improves estimates of when fish transition between geographic locations. Using an empirical Bayesian approach, we show how directional information can be incorporated into the AS modeling framework through the use of informative priors. Our approach refines estimates of survival and movement and allows the AS modeling framework to be applied to new systems with complex geography such as sounds and estuaries. We describe collective migration patterns of acoustic-tagged Pacific herring based on detections at a series of acoustic receiver arrays, deployed at strategic locations throughout the Sound, to showcase the benefits of our modeling approach.

Previously in the Sound, we used acoustic telemetry to investigate post-spawn movements of herring and found that 62% of the 69 acoustic-tagged herring moved to six acoustic arrays located at the entrances of the marine passageways (hereafter referred to as the entrance arrays) that connect the Sound to the Gulf of Alaska (Gulf). During the following fall, we also observed pulses of tagged herring at one of the entrance arrays, suggesting herring were returning from the Gulf. However, the single-line configuration of the entrance arrays prevented confirmation that herring were migrating out into and returning from the Gulf [27]. In 2017, a second line of receivers was added at each of the entrance arrays that made possible the determination of movement direction.

Here we present results from acoustic-tagging Pacific herring while on the spawning grounds in the Sound during spring 2017 and 2018. We hypothesized that if herring were to migrate seasonally between the Sound and the Gulf the movement probabilities from the entrance arrays back into the Sound and from the entrance arrays out into the Gulf would each oscillate seasonally and would alternate in magnitude by season. We used these same movement probabilities to determine if there were seasonal differences in the use of entrance arrays. Finally, we hypothesized that neither physical characteristics of the fish (including length, weight, and sex) nor tag-burden influenced herring movement and survival rates.

## Methods

### Tagging procedures

**Fig. 1:**
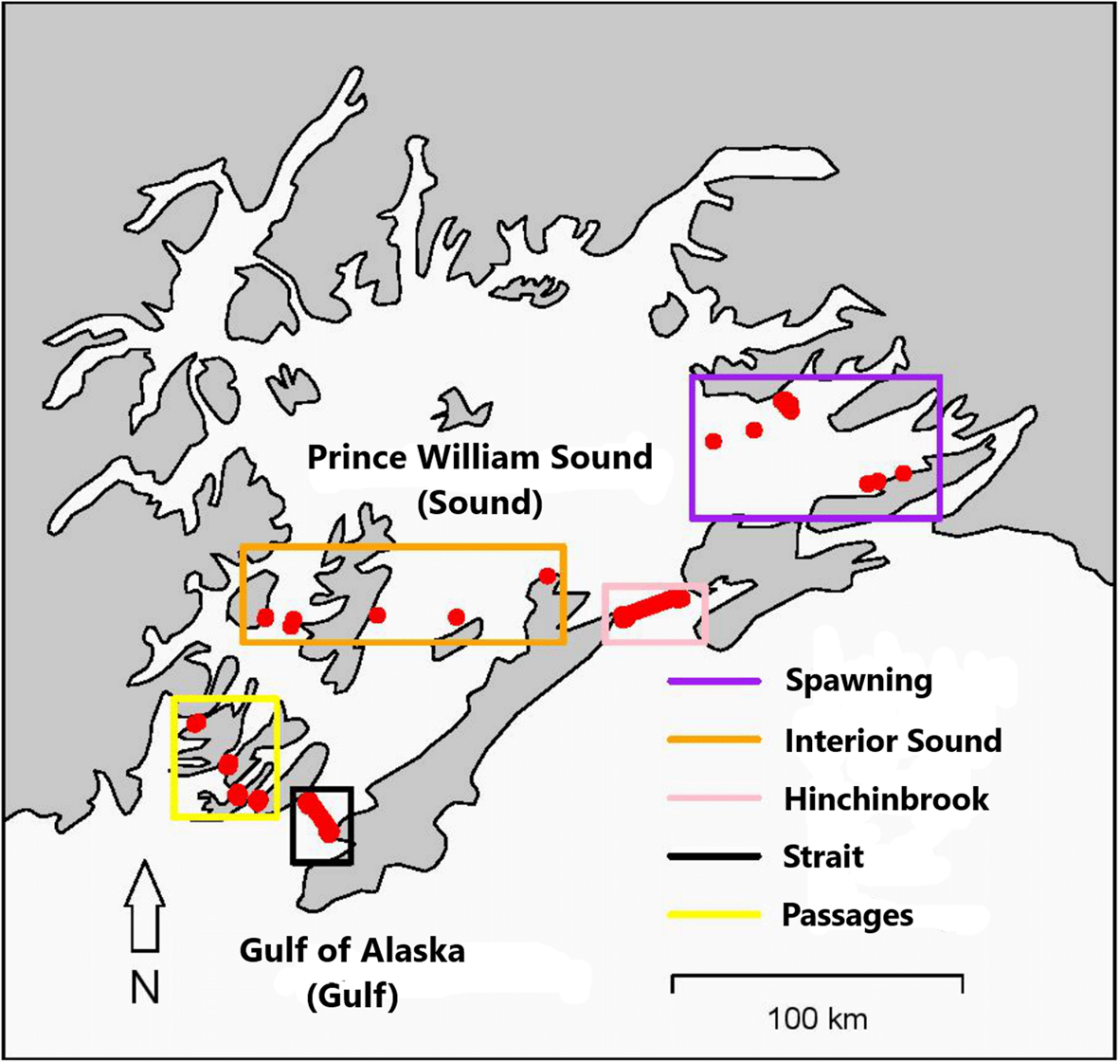
Map of Prince William Sound (the Sound) showing the location of the acoustic receivers (red circles), the spawning area, additional interior arrays, and the three principal passageways into the Sound from the Gulf of Alaska (the Gulf)

We captured and tagged adult Pacific herring during April 2017 (n = 124) and 2018 (n = 202) in the vicinity of and around spring spawning areas in the eastern Sound (Fig. 1). Details regarding the methods to capture and tag the fish have been described previously [3]. Briefly, fish were captured while in prespawning aggregations, anesthetized, and then weighed to the nearest 0.1 g, and measured (standard length, mm). We made a small incision along the ventral midline of the fish to determine sex and surgically implant an acoustic transmitter (Models V9-2x or V8-4x, 69 kHz; Vemco, Halifax, Nova Scotia, Canada). Operational life of the transmitters was estimated at 246 d (Model V8-4x; n=60) and 755-763d (Model V9-2x; n=266). Post-surgery, both tagged and untagged (i.e. not sedated, measured, or tagged) herring from the capture event were held together in a tank to determine when tagged herring had recovered from sedation and exhibited normal swimming and schooling behavior. Tagged and untagged herring were released together.

### Tracking procedures

To monitor the fish we tethered acoustic receivers (Models VR2W, VR2AR, VR3, and VR4; Vemco, Halifax, Nova Scotia, Canada) to stationary moorings on the ocean floor at depths ranging from 3 to 359 m and with distances between adjacent receivers ranging from 378 to 835 m. Fish swimming within reception range of a receiver were detected and the date, time, and unique identity of the fish recorded. Previous range tests for the V9, 145 dB transmitters found that signals were regularly detected (>88% of the time) at distances between 200 and 400 m from receivers [3]. We assumed that detection rates were similar, but slightly less with the V8, 144 dB transmitters. Depending on the year, a total of 59 to 65 receivers were deployed throughout the Sound (Fig. 1), including up to 10 receivers within known spawning grounds in the eastern Sound, and five receivers within the inside waters of the Sound (hereafter referred to as interior Sound receivers).

At the six major entrances connecting the Sound to the Gulf, receivers were arranged in two parallel lines so that information related to the direction of fish movement was recorded. Coverage included 14 receivers at the four southwest passages (Passages), 15 at Montague Strait (Strait), and 20 receivers at Hinchinbrook Entrance (Hinchinbrook). These six arrays located in the Passages, the Strait, and Hinchinbrook collectively comprise the entrance arrays. A 2013 pilot study found that 96–100% of the initial detections at Hinchinbrook and the Strait arrays occurred at the receivers closest to the shoreline [27]. For this reason, we also installed a partial parallel line consisting of two receivers on both sides of Hinchinbrook and the Strait (Fig. 1).

Data were downloaded from the receivers periodically. In our analysis we considered all detections occurring over the 24-month period beginning April 2017. We also assumed that an acoustic-tagged fish was present at an array when two or more detections occurred with a 24 h period.

### Statistical methods

#### The likelihood

In developing the mathematical framework of the likelihood, we begin by indexing individual fish tagged by *i*=1,2,⋯,*m*=326. Let M denote the set of all fish indexes, *c*_*i*_ be the release time of the *i*^*th*^ fish, and *l*_*i*_ be the estimated transmitter life for the *i*^*th*^ fish where *c*_*i*_=0 when the *i*^*th*^ fish was the first to be released into the system. We assumed that an individual’s transmitter is active for the duration of the manufacturer’s estimated battery life. With this assumption the detection periods for which the transmitter is active can be represented by *N*_*i*_={*c*_*i*_+1,*c*_*i*_+2,⋯,*c*_*i*_+*l*_*i*_}. Finally, we let *f* be the final possible detection period corresponding to the 31 March 2019 end date. The AS modeling framework requires a discrete number of detection (or capture) occasions. The fastest fish moved from the spawning receivers to Hinchinbrook in roughly four days; however, it took longer (>2 weeks) for the majority of fish that moved between the two arrays. In order to accurately capture the large scale (i.e. seasonal) movements of the fish while satisfying computational constraints, one-week detection periods were used.

**Fig. 2:**
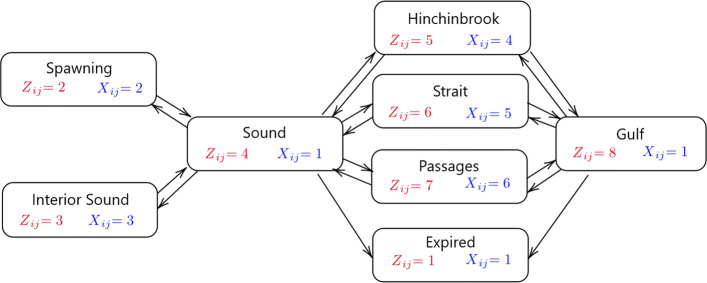
Schematic of model. Arrows pointing to the expired state represent mortality probabilities. All other arrows between nodes illustrate movement probabilities between states. For each node, model states are denoted by *Z*_*ij*_ while model detection events are denoted by *X*_*ij*_

Let *Z*_*ij*_ be a state variable where *i*∈*M* and *j*∈*N*_*i*_∪{*c*_*i*_}, and let the state space be labeled as *S*={1,2,3,4,5,6,7,8}. Referring the reader to Fig. 2, we define *Z*_*ij*_=1 to be the event that the *i*^*th*^ fish has expired at detection period *j*; *Z*_*ij*_=2 that the fish is present at a Sound spawning ground receiver; *Z*_*ij*_=3, that the fish is present at an interior Sound receiver; *Z*_*ij*_=4, that the fish is present within the Sound but not present at a receiver; *Z*_*ij*_=5, that the fish is present at a Hinchinbrook receiver; *Z*_*ij*_=6, that the fish is present at a Strait receiver; *Z*_*ij*_=7, that the fish is present at a Passages receiver; and *Z*_*ij*_=8, that the fish is present in the Gulf.

Let *X*_*ij*_ be a detection variable where *i*∈*M* and *j*∈*N*_*i*_. The detection variables differ from the state variables in a fundamental way. While a tagged fish is always assigned to a state, detections are only possible when a fish passes within the detection range of a receiver. No detections of an acoustic-tagged fish can occur under three scenarios: 1) the fish has expired; 2) the fish is present in the inside waters of the Sound but not within range of any receivers; or, 3), the fish is present in the Gulf. To encode this information, the observation space is defined to be *T*={1,2,3,4,5,6} where *X*_*ij*_=1 is the event where no detection is made for the *i*^*th*^ fish during detection period *j*; *X*_*ij*_=2, that the fish is detected at a spawning grounds receiver; *X*_*ij*_=3, that the fish is detected at an interior Sound receiver; *X*_*ij*_=4, that the fish is detected at a Hinchinbrook receiver; *X*_*ij*_=5; that the fish is detected at a Strait receiver; and *X*_*ij*_=6, that the fish is detected at one the Passages receivers. The scheme used to encode the state and detection variables is detailed further in Fig. 2.

According to the AS modeling framework, when the individual fish are assumed to act independently of one another, the joint likelihood of **Z** and **X** can be factored as

1 $$ {}p(\mathbf{z},\mathbf{x})= \prod_{i \in M}p(z_{ic_{i}})\prod_{j \in N_{i}}p(z_{ij}|z_{i(j-1)})p(x_{ij}|z_{ij})\mathrm{I}(j \leq f)  $$

In this equation, $p(z_{ic_{i}})$ describes the initial state of the *i*^*th*^ fish. Here, we let $P(X_{ic_{i}}=2)=1.$ That is, the initial state of a fish corresponds to its release on the Sound spawning grounds. Additionally, *p*(*z*_*ij*_|*z*_*i*(*j*−1)_) describes how the current state of a fish relates to its previous state, and *p*(*x*_*ij*_|*z*_*ij*_) relates the current state of a fish to its detection data. We define *p*(*z*_*ij*_|*z*_*i*(*j*−1)_) and *p*(*x*_*ij*_|*z*_*ij*_) further in the next section. The indicator variable I(*j*≤*f*) essentially states that the model only considers the detections that occurred before April 2019.

#### Model parameters

Here, we parameterized the model in order to capture the geography of the Sound and Gulf regions and the seasonal movement of herring between these regions. The model is parameterized according to the notation presented by Dupuis and others [38]. Let $\phi _{ij}^{rs}$ and $p_{ij}^{rt}$ be transition and emission (detection) probabilities, respectively. Let *r*,*s*∈*S* and *t*∈*T*. Formally, we define $\phi _{ij}^{rs}$ and $p_{ij}^{rt}$ as $\phi _{ij}^{rs}=P\left (Z_{i(j+1)}=s|Z_{ij}=r\right)$, and $p_{ij}^{rt}=P(X_{ij}=t|Z_{ij}=r)$. Then $\phi _{ij}^{rs}$ is the probability the *i*^*th*^ fish is alive and in state *s* at time *j*+1 given that it was alive and in state *r* at time *j*. The parameter $p_{ij}^{rt}$ is the probability that the *i*^*th*^ fish is detected as *t* at time *j* given that it is in state *r* at time *j*. Two additional parameters (*ψ* and *S*) are introduced into the model in order to distinguish survival from movement. The movement parameter $\psi _{ij}^{rs}$ is interpreted as the conditional probability that the *i*^*th*^ fish in state *r* at time *j* is in state *s* at time *j*+1 given that the fish is alive at time *j*+1. The survival parameter $S_{ij}^{r}$ is interpreted as the probability that the *i*^*th*^ fish survives to time *j*+1 given that the fish is alive and in state *r* at time *j*. To separate movement and survival it is assumed that $\phi _{ij}^{rs}=S_{ij}^{r} \psi _{ij}^{rs}$.

Additional assumptions are added to the model in order to capture the geographic region of interest, to decrease the computational expense of estimating parameters, improve the fit of the model, and to increase the robustness of the parameter estimates. We made two assumptions regarding transition probabilities: fish only expire in the Gulf or within the Sound but not within reception range of a receiver array and survival and movement probabilities are binned by season. Spring and summer season is defined as April through August and fall and winter season as September through March. Spring and summer season corresponds to when herring spawn and then move to feeding grounds; fall and winter season are a period when herring move to deeper overwintering grounds while their gonads ripen [39]. We also assumed that emission probabilities do not depend upon the individual fish or detection time and that an undetected fish is in the Sound but away from receivers, or is in the Gulf, or has expired. Finally, we assumed an acoustic-tagged fish is always detected when it passes by an array. Because a series of receivers are located at each array and most fish move along the sides of the Sound’s passages which are lined with two sets of receivers, there is a high probability of fish detection [3].

The transition and emission probabilities can be expressed as matrices (referred to as transition and emission matrices respectively). For the *i*^*th*^ fish at detection period *j*, the transition matrix is defined to be $T^{(ij)}_{rs}=\phi _{ij}^{rs}$ and the emission matrix to be $E^{(ij)}_{tr}=p_{ij}^{rt}$. Supposing that there are M seasons and that *B*_*k*_ is the set of all detection times in the *k*^*th*^ season, the transition and emission matrices for the *i*^*th*^ fish at detection period *j* are defined to be

$$\begin{array}{*{20}l} T^{(ij)} &= \left\{\begin{array}{cccccccc} 1 & 0 & 0 & \phi_{ik}^{14} & 0 & 0 & 0 & \phi_{ik}^{18} \\ 0 & \phi_{ik}^{22}& 0 & \phi_{ik}^{24} & 0 & 0 & 0 & 0 \\ 0 & 0 & \phi_{ik}^{33} & \phi_{ik}^{34} & 0 & 0 & 0 & 0 \\ 0 & \phi_{ik}^{42}& \phi_{ik}^{43} & \phi_{ik}^{44} & \phi_{ik}^{45} & \phi_{ik}^{46} & \phi_{ik}^{47} & \phi_{ik}^{48}\\ 0 & 0 & 0 & \phi_{ik}^{54} & \phi_{ik}^{55} & 0 & 0 & \phi_{ik}^{58} \\ 0 & 0 & 0 & \phi_{ik}^{64} & 0 & \phi_{ik}^{66} & 0 & \phi_{ik}^{68} \\ 0 & 0 & 0 & \phi_{ik}^{74} & 0 & 0 & \phi_{ik}^{77} & \phi_{ik}^{78}\\ 0 & 0 & 0 & 0 & \phi_{ik}^{85 }& \phi_{ik}^{86} & \phi_{ik}^{87} & \phi_{ik}^{88} \end{array}\right\}\\ E^{(ij)}&= \left\{\begin{array}{cccccccc} 1 & 0 & 0 & 1 & 0 & 0 & 0 & 1 \\ 0 & 1 & 0 & 0 & 0 & 0 & 0 & 0 \\ 0 & 0 & 1 & 0 & 0 & 0 & 0 & 0 \\ 0 & 0 & 0 & 0 & 1 & 0 & 0 & 0 \\ 0 & 0 & 0 & 0 & 0 & 1 & 0 & 0 \\ 0 & 0 & 0 & 0 & 0 & 0 & 1 & 0 \end{array}\right\} \end{array} $$

where *j*∈*B*_*k*_. In understanding the movement and survival dynamics of the fish, we first considered *M*=4 seasons (two spring and summer and two fall and winter). Here, *B*_1_ was defined to be the set of detection times ranging from April through August 2017; *B*_2_, September 2017 through March 2018; *B*_3_, April through August 2018; and *B*_4_, September 2018 through March 2019. The transition matrix was developed to reflect the model schematic in Fig. 2.

In our modeling framework, tag shedding cannot be distinguished from mortality. Additionally, mortality cannot be distinguished from permanent immigration to the Gulf. Because mortality cannot be distinguished from permanent immigration in the Gulf, mortality rates in the Gulf are positively biased. However, as the rates at which herring migrate permanently into the Gulf is expected to be relatively low, we refer to the Gulf mortality and immigration rates as the Gulf mortality rates.

#### Determining the factors that influence survival and movement

A linear constraint was incorporated into the model to understand which factors influence the survival and movement rates. Here, a logit link function was used to model the movement and survival probabilities as a function of a categorical variable. The variables considered were standard length, weight, condition (weight×length^−3^), sex, and tag-burden (tag weight/fish weight). The median was used as the break point in separating the variable of interest into two levels. In the weight model, we let *y*_*i*_ be an indicator variable where *y*_*i*_=0 when the *i*^*th*^ fish weighs less than or equal to the median weight and *y*_*i*_=1 when the *i*^*th*^ fish weighs more than the median weight. The survival probabilities are now modeled as $\text {logit}\left (S_{ik}^{r}\right)=\beta _{0k}^{r}+\beta _{1k}^{r}y_{i}$ and the movement probabilities are modeled as $\text {logit}\left (\psi _{ik}^{rs}\right)=\beta _{0k}^{rs}+\beta _{1k}^{rs}y_{i}$. Analogous to a simple linear regression, the parameters $\beta _{1 k}^{r}$ and $\beta _{1 k}^{r s}$ can be used to determine if the variables length, weight, condition, sex, or tag-burden influence herring survival and movement. Because the survival and movement probabilities do not depend upon the individual fish, $S_{y_{i}k}^{rs}$ and $\psi _{y_{i}k}^{rs}$ will henceforth be used to denote $S_{ik}^{rs}$ and $\psi _{ik}^{rs}$. We built five constrained models, one for each covariate. Additionally, we built an unconstrained version of the model with no covariates. We drop the subscript *i* when referring to parameters within this unconstrained model as the survival and movement probabilities do not depend upon characteristics of individual fish.

To determine if mortality increased during the initial months following the tagging procedure, two different variations of the model were run. In the first variation, fish tagged in 2017 were used to estimate the mortality rates in the Sound and the Gulf during the first and second half of the 2017 spring and summer season; in the second variation, fish tagged in 2018 were used to estimate the mortality rates in the first and second half of the 2018 spring and summer season. If fish were to expire immediately after the tagging procedure, the mortality rates would be higher during the first half of both the 2017 and 2018 spring and summer.

### Priors

If some information about a model parameter is known before the start of an experiment, a prior density reflecting the degree of subjective belief about the parameter can be selected so that the posterior density is a compromise between the likelihood and the prior. On the other hand, if no information is known about a model parameter at the start of an experiment, an uninformative prior can be selected. Because fine scale directional data recorded at the six entrance arrays were not considered in developing the likelihood, an informative prior was placed on the transition probabilities at the entrance arrays (Hinchinbrook, the Strait, and the Passages) to incorporate the directional data. For the Sound spawning grounds and the interior Sound receivers, non-informative priors are placed on the transition probabilities as directional information is not recorded at these locations. Non-informative priors are also placed on the survival probabilities in the Sound and the Gulf.

#### Informative priors at the entrance arrays

To determine the passage direction associated with detections made at each of the six entrance arrays, we used an algorithm to determine the first and last receiver(s) where detections occurred. The algorithm considers a fish to move from the Sound to the Gulf if the fish is detected by an entrance array’s inner line of receivers first and the outer line last; a fish is considered to move from the Gulf to the Sound if the fish is detected by the entrance array’s outer line first and the inner line last. In our implementation, a fish was considered to have left the entrance array if it was undetected for a 24-h period after the last detection.

**Fig. 3:**
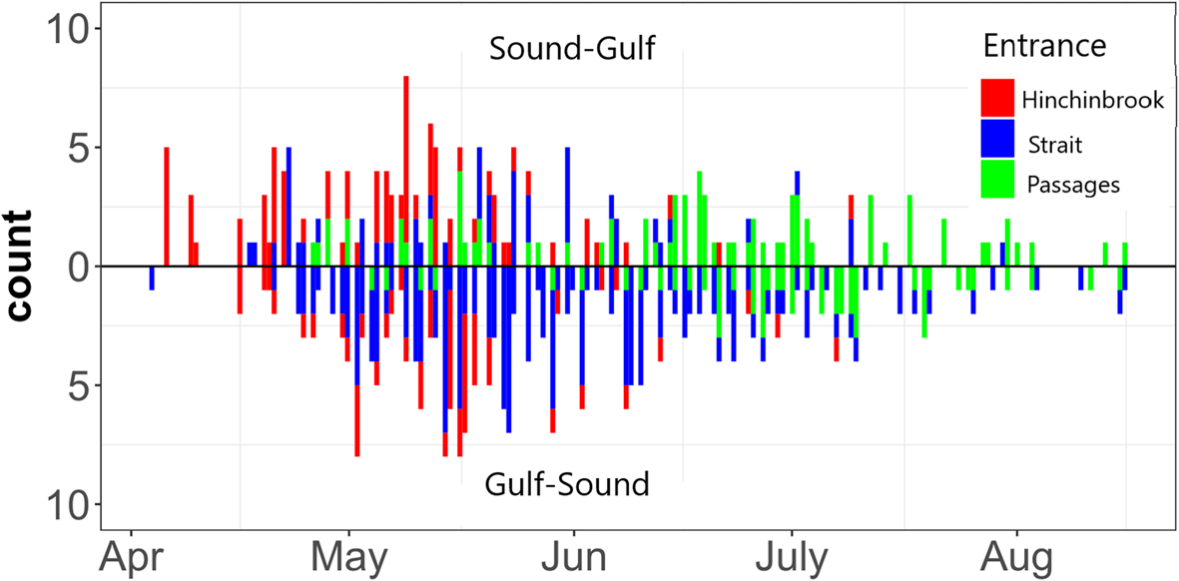
Directional detections at the three arrays situated at the entrances between the Sound and the Gulf, April-August 2018. The histogram depicts the detections at Hinchinbrook, the Strait, and the Passages where our algorithm assigned a direction

At Hinchinbrook and the Strait arrays, only the receivers closest to the east and west shorelines had both inner and outer line receivers. Therefore, passage direction was not assigned when a fish was detected at inner line receivers that lacked a complementary outer line. In addition, passage direction was not assigned when the first or last detections coincided between inner and outer line receivers. Inner and outer line detections coincided <10*%* of all detections. A histogram of the April–August 2018 detections at the entrance arrays where passage direction was assigned is depicted in Fig. 3.

In defining the informative priors at the entrance arrays, let *y* represent a particular fish class. For example, *y*=0 may represent the class of fish with weights less than or equal to the median weight of the tagged fish. Now let $d_{yk}^{r8}$ be the number of well-defined Sound to Gulf directional detections for the *y*^*th*^ class during the *k*^*th*^ season at the *r*^*th*^ entrance array, and let $d_{yk}^{r4}$ be the number of well-defined Gulf to Sound directional detections for the *y*^*th*^ class during the *k*^*th*^ season at the *r*^*th*^ entrance array. Here, *r*∈{5,6,7} where *r*=5 corresponds to Hinchinbrook, *r*=6 to the Strait, and *r*=7 to the Passages. Let *q*_*r*_ be the number of fish that stayed at the *r*^*th*^ entrance array divided by the number of fish that left the entrance array between consecutive detection occasions. We then estimate the number of fish in the *y*^*th*^ class that stay at the *r*^*th*^ entrance between consecutive detection periods relative to the number of fish that leave as $d_{yk}^{rr}=q_{r}\left (d_{yk}^{r4}+d_{yk}^{r8}\right)$. The counts $\left (d_{yk}^{r4}, d_{yk}^{rr}, d_{yk}^{r8}\right)$ can be seen as arising from a $\text {Multinomial}\left (\psi _{yk}^{r4}, \psi _{yk}^{rr}, \psi _{yk}^{r8}\right)$ distribution; therefore, in order to incorporate the fine-scale directional information, the prior densities for the movement probabilities at the entrance arrays are distributed as

$$\left(\psi_{yk}^{r4}, \psi_{yk}^{rr}, \psi_{yk}^{r8}\right) \thicksim \text{Dirichlet}\left(d_{yk}^{r4}+1, d_{yk}^{rr}+1, d_{yk}^{r8}+1\right). $$

Here, the informative Dirichlet priors placed on the movement probabilities at the entrance arrays incorporate directional fish count information into the model.

#### Non-Informative priors

Because directional information was not recorded at the receiver arrays located at either the Sound spawning grounds or the interior Sound, non-informative prior densities were placed on the remaining movement probabilities. Here, we continue with the Dirichlet family of distribution. Specifically, the prior distributions on the movement probabilities at: Sound spawning grounds arrays, Sound interior arrays, areas within Sound waters but away from the arrays, and Gulf waters are defined as

$$\begin{array}{*{20}l} &\left(\psi_{yk}^{22},\psi_{yk}^{24}\right) \thicksim \text{Dirichlet}(2,2)\\ &\left(\psi_{yk}^{33},\psi_{yk}^{34}\right) \thicksim \text{Dirichlet}(2,2)\\ &\left(\psi_{yk}^{42},\psi_{yk}^{43},\psi_{yk}^{44},\psi_{yk}^{45},\psi_{yk}^{46},\psi_{yk}^{47}\right) \thicksim \text{Dirichlet}(2,2,2,2,2,2)\\ &\left(\psi_{yk}^{85},\psi_{yk}^{86},\psi_{yk}^{87},\psi_{yk}^{88}\right) \thicksim \text{Dirichlet}(2,2,2,2) \end{array} $$

Non-informative Beta priors were placed on the survival probabilities in the Sound and in the Gulf. Here,

$$\begin{array}{*{20}l} & S_{yk}^{4} \thicksim \text{Beta}(2,2)\\ & S_{yk}^{8} \thicksim \text{Beta}(2,2) \end{array} $$

The product of the previously defined informative and not-informative priors defines the joint prior density.

### Computation and convergence diagnostics

The entire analysis was carried out using the programming language R [40]. For the Markov Chain Monte Carlo (MCMC) step, the Gibbs sampler JAGS [41] and R interface Rjags [42] were used to approximate the posterior density. Four chains were run for 200,000 iterations each with a burn-in period of 10,000 iterations and a thinning period of 25 iterations. Trace and Gelman-Rubin-Brooks plots [43] were used to ensure that the chains had converged. Different variations of the model were run and the Deviance Information Criterion (DIC) was used as the metric to compare the fit of the models.

## Results

We obtained 322,258 detections at the receivers over the 24-month study period. A total of 178,284 of these detections occurred at the spawning ground arrays, 2,506 detections at the interior Sound arrays, and 141,468 detections at the entrance arrays. Most of the entrance array detections came from the Strait (54,044 detections) and the Passages (56,820 detections). Of the 124 herring tagged in 2017, 1 was never detected, 64 were detected at spawning grounds receivers only, and 59 were detected at receivers outside of the spawning grounds; of the 202 herring tagged in 2018, 17 were never detected, 48 were detected at spawning grounds receivers only, and 137 were detected at receivers outside of the spawning grounds. Fish were detected at Hinchinbrook, the Strait, and the Passages arrays on 397, 611, and 178 separate occasions, respectively.

### Seasonal migration between the sound and the gulf

We determined seasonal migration patterns of the tagged herring using the unconstrained model. If a portion of the tagged herring were to migrate seasonally between the Sound and the Gulf, both the entrance arrays to the Sound and the entrance arrays to the Gulf movement probabilities ($\psi _{j}^{r4}$ and $\psi _{j}^{r8}$ with *r*∈{5,6,7}) would oscillate seasonally (eg $\psi _{1}^{r4}, \psi _{3}^{r4} > \psi _{2}^{r4}, \psi _{4}^{r4}$ and $\psi _{1}^{r8}, \psi _{3}^{r8} < \psi _{2}^{r8}, \psi _{4}^{r8}$). Additionally, probabilities for the entrance arrays to the Sound and entrance arrays to the Gulf would alternate in magnitude by season (eg $\psi _{1}^{r4} > \psi _{1}^{r8}, \psi _{2}^{r4} < \psi _{2}^{r8}, \psi _{3}^{r4} > \psi _{3}^{r8}$, and $\psi _{4}^{r4} < \psi _{4}^{r8}$). This trend is especially important as it distinguishes seasonal migration from non-seasonal movement.

**Fig. 4:**
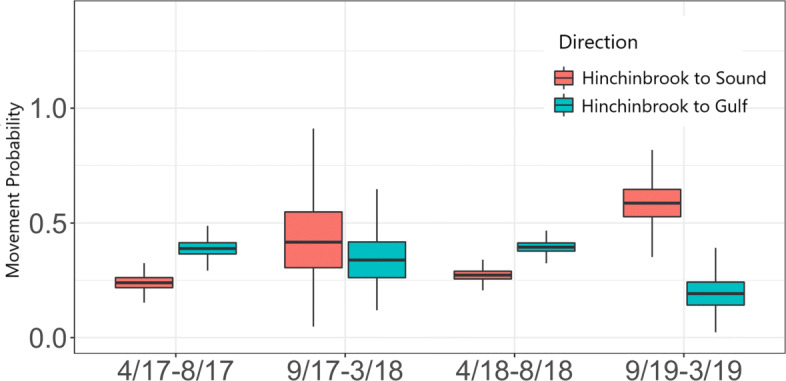
Pacific herring seasonal movement probabilities between Hinchinbrook and the Sound ($\psi _{j}^{54}$) and between Hinchinbrook and the Gulf ($\psi _{j}^{58}$). Probabilities are based on weekly time periods. The Hinchinbrook to Sound and the Hinchinbrook to Gulf movement probabilities oscillate by season. During the spring and summer season (April-August) fish are more likely to move from the Sound to the Gulf than from the Gulf to the Sound. During the fall and winter season (September-March) the trend reverses

We confirmed these oscillating patterns in the movement probabilities at one of the three entrances. At Hinchinbrook during the spring and summer the rate at which herring pass into the Gulf was higher than the rate at which herring passed into the Sound, whereas the opposite trend was reflected during fall and winter (Fig. 4). However, this pattern was not observed in the movement probabilities at the Strait or the Passages arrays (Table 1).

**Table 1:** Pacific herring movement probabilities at Hinchinbrook, the Strait, and the Passages. The table details the full set of movement probabilities between the entrance arrays and the Sound ($\psi _{j}^{r4}$) and between the entrance arrays and the Gulf ($\psi _{j}^{r8}$). A seasonal back and forth pattern is found at Hinchinbrook. However, this pattern is not found at the Strait or the Passages

Location	Weekly probability of movement (median estimate; 90% CI)			
from-to	4/17-8/17	9/17-3/18	4/18-8/18	9/19-3/19
Hinchinbrook-Sound	0.24; (0.19, 0.30)	0.42; (0.17, 0.72)	0.27; (0.24, 0.32)	0.59; (0.44, 0.73)
Hinchinbrook-Gulf	0.39; (0.33, 0.45)	0.34; (0.10, 0.63)	0.39; (0.35,0.44)	0.19; (0.09, 0.32)
Strait-Sound	0.18; (0.14,0.24)	0.23; (0.18,0.27)	0.36; (0.33,0.39)	0.13; (0.11,0.16)
Strait-Gulf	0.37; (0.31,0.43)	0.21; (0.17,0.26)	0.12; (0.10,0.14)	0.39; (0.36,0.43)
Passages-Sound	0.22; (0.15,0.31)	0.25; (0.16,0.35)	0.21; (0.18,0.25)	0.31; (0.17,0.49)
Passages-Gulf	0.35; (0.26,0.45)	0.32; (0.22,0.42)	0.29; (0.26,0.33)	0.33; (0.18,0.50)

To determine if there was a seasonal difference in the use of entrance arrays, we calculated movement probabilities between the Sound and the entrance arrays and between the Gulf and the entrance arrays ($\psi _{j}^{4r}$ and $\psi _{j}^{8r}$ with *r*∈{5,6,7}). During the spring and summer season herring entered the Gulf primarily through Hinchinbrook whereas, during the fall and winter season herring returned to the Sound from the Gulf primarily through the Strait (Table 2). An exception was in the fall and winter season of 2017/2018 when a large portion of fish moved from the Gulf to the Passages during September.

**Table 2:** Seasonal movement probability point estimates and credible intervals for acoustic tagged Pacific herring moving between the Sound and the entrance arrays $\left (\psi _{j}^{4r}\right)$ and between the Gulf and the entrance arrays $\left (\psi _{j}^{8r}\right)$. The entrance arrays include those in Hinchinbrook, the Strait, and the Passages. During spring and summer season (April–August) fish move from the Sound to the Gulf primarily through Hinchinbrook. During the fall and winter months (September—March) fish move from the Gulf to the Sound through the Strait

Date	Location	Weekly probability of movement
(month/year)	(from-to)	(median estimate; 90% CI)
4/17 to 8/17	Sound-Hinchinbrook	0.09; (0.07, 0.12)
4/17 to 8/17	Sound-Strait	0.05; (0.04, 0.07)
4/17 to 8/17	Sound-Passages	0.05; (0.04, 0.07)
4/18 to 8/18	Sound-Hinchinbrook	0.10; (0.08, 0.11)
4/18 to 8/18	Sound-Strait	0.05; (0.04, 0.06)
4/18 to 8/18	Sound-Passages	0.05; (0.04, 0.06)
9/17 to 3/18	Gulf-Hinchinbrook	0.02; (0.01, 0.04)
9/17 to 3/18	Gulf-Strait	0.05; (0.03, 0.08)
9/17 to 3/18	Gulf-Passages	0.05; (0.03, 0.07)
9/18 to 3/19	Gulf-Hinchinbrook	0.02; (0.01, 0.03)
9/18 to 3/19	Gulf-Strait	0.09; (0.07, 0.11)
9/18 to 3/19	Gulf-Passages	0.01; (0.00, 0.01)

The constrained model was run separately on each variable of interest (length, weight, condition, sex, and tag-burden). Length, weight, and condition were each found to have a significant effect on the rate at which herring move from the Sound to the entrance arrays. Sex and tag burden did not have a significant effect on the rates of herring movement. For brevity, we have chosen to summarize the results for the weight variable only. Here, heavier fish were more likely to move from the Sound to the Strait entrance during the spring and summer season (90% CIs: $\beta _{11}^{46}$ [-1.24, -0.02]; $\beta _{13}^{46}$ [-1.43, -0.14]) while lighter fish were shown to be more likely to move from the Sound to the spawning grounds arrays during the fall and winter season (90% CIs: $\beta _{12}^{42}$ [0.51, 2.15]; $\beta _{14}^{42}$ [0.44, 1.37]).

### Survival

**Fig. 5:**
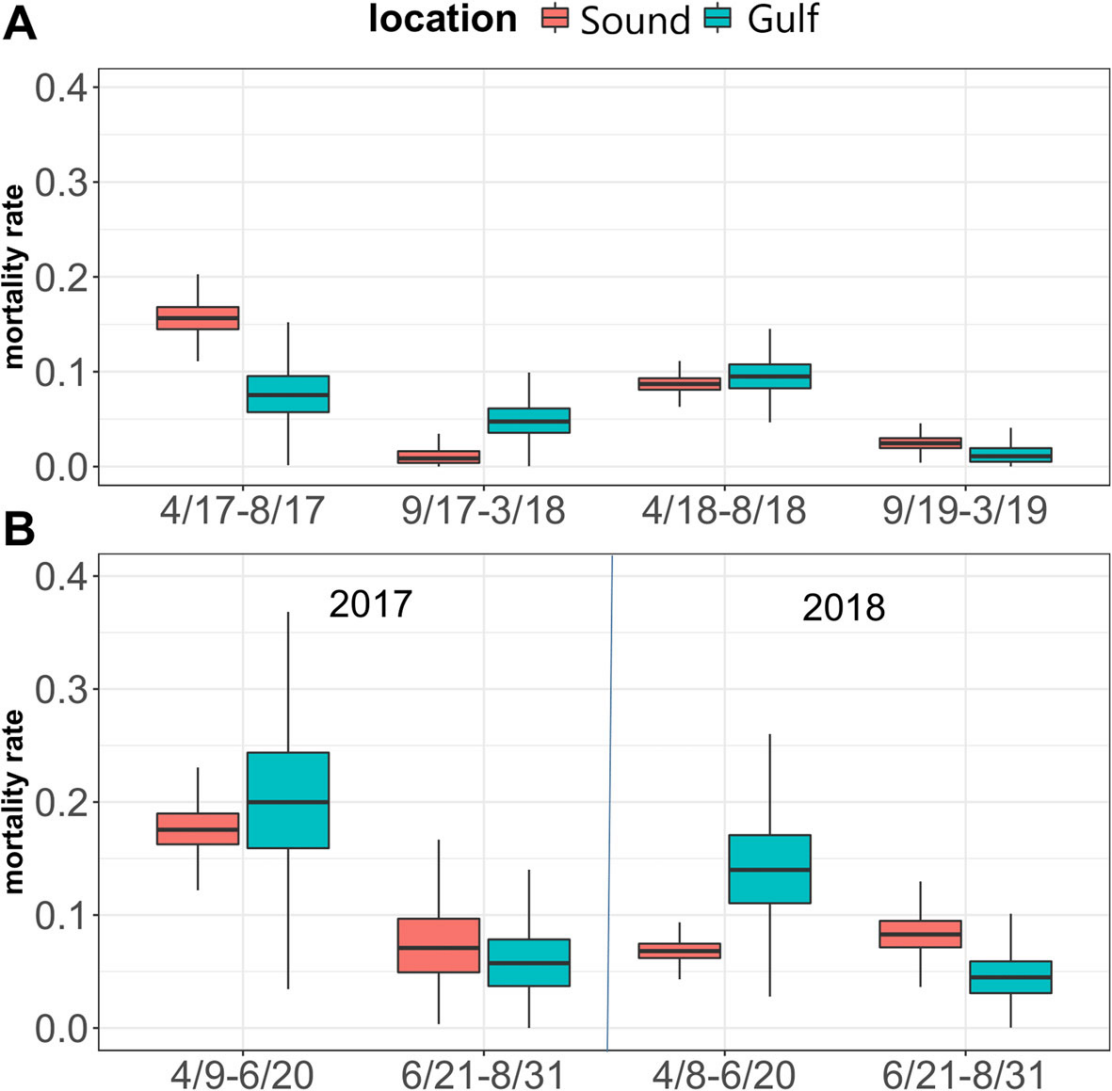
**a** Seasonal mortality rates for Prince William Sound Pacific herring based on weekly time periods. Mortality rates in the Sound ($1-S_{j}^{4}$) are denoted in red. Mortality rates in the Gulf ($1-S_{j}^{8}$) are denoted in blue. Mortality was higher during the spring and summer season (April-August) than the fall and winter season (September-March) in both the Sound and the Gulf. **b** Mortality rates for Pacific herring tagged in 2017 and 2018 during the initial months following the tagging procedure

The unconstrained model was used to estimate the seasonal mortality rates in the Sound ($1-S_{j}^{4}$) and Gulf ($1-S_{j}^{8}$). These parameters were not found to differ significantly from each other. Because the estimated mortality rate in the Gulf is positively biased, true mortality was not found to be higher in the Sound than in the Gulf. In the Sound, mortality was significantly higher during the spring and summer than the fall and winter. In the Sound, the weekly mortality rate was estimated to be 0.16 and 0.09 in the spring/summer of 2017 and 2018 compared to 0.01 an 0.03 in the fall and winter of 2017 and 2018. In the Gulf, the median point estimates reflect the same seasonal trend as in the Sound: the mortality rates were higher during the spring and summer than the fall and winter (Fig. 5a). Given the lifespan of Pacific herring in the Sound (8–13 years), mortality rates were higher than expected during the spring and summer season. For instance, during spring and summer 2017 the weekly death rate in the Sound was estimated to be 0.15. These trends would appear if mortality was higher for newly tagged fish.

When we analyzed mortality in the initial months following the tagging procedure, our results show that in 2017, mortality was higher in both the Sound and the Gulf during the first half of the spring and summer season compared to the second half of the season. The same trend appeared in the Gulf for the fish tagged in 2018. However, 2018 fish mortality rates in the Sound were similar between the first and second half of the spring and summer season (Fig. 5).

The constrained model was used to investigate the relationship between tag burden and mortality. Tag-burden was not shown to have a significant effect on herring survival in the Sound or in the Gulf (0∈ 90% CIs for $\beta _{2j}^{4}$ and $\beta _{2j}^{8}$ for *j*∈{1,2,3,4}).

## Discussion

Our study shows how the AS modeling framework can be used to specify a likelihood that simultaneously models both seasonal migration between the Sound and the Gulf and survival. Existing software, such as Program MARK [44], could be used to estimate the survival and movement model parameters expressed in our likelihood (Equation 1) using the Expectation Maximization (EM) algorithm. However, with this approach, directional information recorded from the entrance arrays cannot be taken into consideration resulting in less precise parameter estimates. In the case of the Sound herring, when directional information is ignored, the AS model is unable to determine whether a fish moved into the Sound or into the Gulf. One solution would be to define temporally shorter detection events and to add additional model states so that there are separate states for the inner and outer receiver lines at each entrance. However, existing software is not equipped to handle large numbers of detection events, as would be the case here. The problem becomes increasingly complex when the direction of animal movement can be inferred for some but not all detections. With an empirical Bayesian modeling approach, incomplete directional information can be used to inform prior densities which can reduce the degree of state uncertainty to a level that allows researchers to answer questions related to fish survival and movement.

### Success of the tagging procedure

When working with stationary receivers spread across geographically complex regions, it can be difficult to estimate the degree to which a capture and tagging procedure influences fish survival. Numerous studies have used return rates to report varying degrees of success marking herring with external tags [4, 45, 46]. However, when there is a large degree of uncertainty regarding the location of fish, return rates may not reflect fish survival. The AS modeling framework allows researchers to separate movement from survival so that mortality can be estimated directly. Additionally, this framework allows survival to be modeled as a function of time as well as a function of features related to the capture and tagging process such as handling-related stress or tag burden. This is a useful modeling feature in that fish mortality can be attributed to the capture and tagging procedure itself.

We found that mortality rates of herring did not differ by tag-burden category. This does not mean that tag-burden had no influence on fish survival as our two tag-burden categories may not have expressed enough variation to show a significant effect given the power of our model. We did find evidence that the tagging procedure had a differential effect on herring survival over time. Our results suggest that mortality spikes in the initial weeks following the tagging procedure and this rate decays over time.

This result does not come as a surprise. Herring spawning aggregations attract large numbers of predators including seabirds, Steller sea lions (*Eumetopias jubatus*), and humpback whales (*Megaptera novaeangliae*)[47, 48]. In addition, laboratory studies have shown that the tagging procedure can lead to mortality rates as high as 4% in the first four weeks, and tag shedding rates of 4% between 6–8 weeks after surgery [49]. However, the quantification of mortality can yield insight that can be used to enhance sampling efforts. For example, fish mortality was estimated to be higher in the two months following the tagging campaign for fish tagged in 2017 compared to fish tagged in 2018. Environmental variability could explain these differences; nevertheless, our results suggest that the tagging campaign was more successful in 2018.

### Migratory dynamics of sound herring

The lack of recovery by the Pacific herring population in the Sound more than 30 years after the *Exxon Valdez* oil spill, makes it increasingly important to document adult migration patterns and survival. However, determining how herring migrate between spawning, feeding, and wintering areas can be challenging because of technological, logistical, and financial constraints. In this paper we have demonstrated how Bayesian AS models can be used to analyze stationary acoustic telemetry data to gain insight into the movement and migration dynamics of fish. In the case of the Sound herring, the migration pattern found in the movement probabilities at Hinchinbrook show that a portion of the Sound herring population migrates seasonally between the Sound and the Gulf through Hinchinbrook. Our estimates show that these fish tended to enter the Gulf during the spring and summer after spawning and return to the Sound during the fall and winter. We suggest the movements into the Gulf are related to the differences between the Gulf and the Sound in the timing of the spring plankton bloom and the associated increase in large calanoid copepods (primarily *Neocalanus pluchrus* and *N. flemingeri*), which are important herring prey. Satellite imagery and in situ measurements indicated that the plankton bloom occurs earlier in the Sound than along the continental shelf [50].

A migratory pattern did not appear at the Strait or the Passages. A migratory pattern would not be present if herring were taking an indirect path of passing repeatedly back and forth through the Strait and the Passages in route to the wintering grounds. Results from the Passages suggest this is the case. Across both fall and winters of this study, herring were detected at the Passages only during September 2017. These fish were found to be equally likely to move from the Passages into the Sound as into the Gulf.

However, other explanations exist for why the migratory pattern did not appear at the Strait and the Passages. A migratory pattern would not appear if these entrances were located at the far end of the Sound herring population’s migratory range. Additionally, this pattern would not appear if the seasonal definitions (April to August and September to March) were misaligned to capture herring’s back and forth movement at these locations.

Evidence suggests that the Sound’s herring are partial migrants [19]. That is, the Sound’s Pacific herring population consists of both resident and migratory fish. Aerial forage fish surveys conducted during June and July throughout the Sound have noted the persistence of adult herring schools [51], suggesting that areas within the Sound may serve as summer feeding grounds. Furthermore, the major biomass of adult herring currently overwinters close to the spring spawning grounds [52]. However, commercial fishers have reported large schools of herring moving from the Gulf into the Sound during both fall and spring while others have observed herring during winter in nearby Gulf waters [53].

Given that both resident and migratory populations exist within the Sound, our model helps discern the factors that potentially distinguish the two groups. Lighter fish were shown to be more likely to move from the Sound to the spawning grounds arrays in the winter months (they were more likely to stay in the Sound and return to the spawning grounds) while heavier fish were more likely to move from the Sound to the Strait during the spring and summer season. Because weight is positively correlated with length and age, our results suggest that the heavier, longer, and older fish are more likely to migrate than smaller, younger fish. However, weight was not found to have a significant influence on the probability of movement from the Sound to Hinchinbrook during the spring and summer. Because the Strait is located further from the spawning grounds than Hinchinbrook, heavier herring may simply travel greater distances within their seasonal migration than lighter herring.

Similarly, results from field studies of migration in Atlantic herring (*C. harengus*) suggested that migration distance is a function of length, weight, and age, with the extent of migration increasing with increasing body length. In this same study, their model results predicted that for smaller (<20 cm) fish, long-distance migration costs may exceed energy intake, due to increased hydrodynamical drag with decreasing fish size [54].

## Conclusion

As acoustic transmitters become increasingly small, stationary acoustic telemetry is expected to become more commonly used for studying the movement patterns of small fish. It can be challenging to analyze acoustic telemetry data using ordinary AS models if information related to the direction of fish travel is recorded by the receivers. Directional information is especially important when building models over geographically complex regions. We have shown how an empirical Bayesian modeling approach can be used to extend the AS modeling framework to incorporate directional information. Using this approach, we have demonstrated that some of the Sound’s herring population migrate seasonally between the Sound and the Gulf and we documented the timing and direction of this movement. AS models have the added benefit that movement and survival are modeled simultaneously. This is useful as these models can be used to assess the efficacy of a tagging campaign. The modeling framework presented in this document is very general and can be applied to other systems and species.

## Data Availability

The datasets and computer code used during the current study are available from the corresponding author on reasonable request.

## Abbreviations

AS: Arnason-Schwarz
Gulf: Gulf of Alaska
Sound: Prince William Sound
Hinchinbrook: Hinchinbrook Entrance
Strait: Montague Strait
Passages: Southwest Passages
MCMC: Markov Chain Monte Carlo
